# Continuous elevation of lung sound amplitudes, recorded at fixed flow rate, may indicate an increase in lung water content

**DOI:** 10.1186/cc9594

**Published:** 2011-03-11

**Authors:** S Lev, P Singer, K Robinson, K Hojnowski, L Wolloch, L Gatto, GF Nieman

**Affiliations:** 1 Rabin Medical Center, Beilinson Campus, Petach Tikva, Israel; 2 SUNY Upstate Medical University, Syracuse, NY, USA; 3 Deep Breeze Ltd, Or-Akiva, Israel; 4 SUNY Cortland, Cortland, NY, USA

## Introduction

Vibration response imaging (VRI) is a bedside lung sound monitoring system. We previously reported that vibration intensity can be significantly elevated in patients with congestion, as opposed to pleural effusion, atelectasis, or normal lung [1]. We hypothesized that changes in lung water content (that is, pulmonary edema) may influence breath sound amplitude and explored the possibility of using continuous digitalized lung sound monitoring as a means to track changes in extravascular lung water (EVLW).

## Methods

EVLW was increased in three pigs: in two animals by installation of saline into the endotracheal tube, and in one animal with sepsis-induced edema. In both models the increase in extravascular lung water index (EVLWi) was evaluated by the PiCCO system, and lung sound amplitude was monitored with the VRI. Animals were ventilated at a fixed flow rate.

## Results

**Figure 1:**
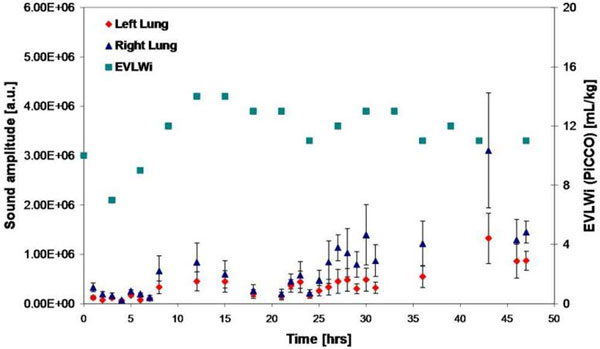
**Sound intensity and EVLWi versus time, in a septic pig model (average ± SEM)**.

In both the saline installation and sepsis animal models, significant elevation in lung sound amplitude was measured. In the saline installation animals, sound amplitude increased from 2.21 × 10^5 ^± 1.58 × 10^4 ^au to 9.49 × 10^5 ^± 8.02 × 10^4 ^au (average ± SEM), concomitant with an increase in EVLWi from 10 ml/kg to 14 ml/kg. Similarly, sound amplitudes changed in correspondence with elevation of EVLWi in the septic animal (see Figure 1).

## Conclusions

These preliminary results suggest that continuous elevation of lung sound amplitudes, recorded at fixed flow rate, may indicate an increase in lung water content.
